# Zebrafish Cancer Avatars: A Translational Platform for Analyzing Tumor Heterogeneity and Predicting Patient Outcomes

**DOI:** 10.3390/ijms24032288

**Published:** 2023-01-24

**Authors:** Majd A. Al-Hamaly, Logan T. Turner, Angelica Rivera-Martinez, Analiz Rodriguez, Jessica S. Blackburn

**Affiliations:** 1 Pharmacology and Nutritional Sciences, University of Kentucky, Lexington, KY 40356, USA; 2 Markey Cancer Center, University of Kentucky, Lexington, KY 40536, USA; 3 Molecular and Cellular Biochemistry, University of Kentucky, Lexington, KY 40356, USA; 4 Department of Neurosurgery, University of Arkansas for Medical Sciences, Little Rock, AR 72205, USA

**Keywords:** co-clinical trials, intra-tumoral heterogeneity, precision medicine, xenograft, zPDX

## Abstract

The increasing number of available anti-cancer drugs presents a challenge for oncologists, who must choose the most effective treatment for the patient. Precision cancer medicine relies on matching a drug with a tumor’s molecular profile to optimize the therapeutic benefit. However, current precision medicine approaches do not fully account for intra-tumoral heterogeneity. Different mutation profiles and cell behaviors within a single heterogeneous tumor can significantly impact therapy response and patient outcomes. Patient-derived avatar models recapitulate a patient’s tumor in an animal or dish and provide the means to functionally assess heterogeneity’s impact on drug response. Mouse xenograft and organoid avatars are well-established, but the time required to generate these models is not practical for clinical decision-making. Zebrafish are emerging as a time-efficient and cost-effective cancer avatar model. In this review, we highlight recent developments in zebrafish cancer avatar models and discuss the unique features of zebrafish that make them ideal for the interrogation of cancer heterogeneity and as part of precision cancer medicine pipelines.

## 1. Introduction

The field of precision oncology focuses on the molecular characterization of a patient’s tumor, with the goal of identifying actionable and patient-specific mutations that can be exploited therapeutically [1,2,3]. Precision medicine holds great promise in the clinic, but emerging data suggest that treating a cancer patient with targeted therapy is not as simple as matching a particular drug to a tumor mutation. In the National Cancer Institute’s precision medicine trial, called the Molecular Analysis for Therapy Choice (NCI MATCH), more than 5000 patients were assigned to a treatment based on the genetics of their tumor. However, fewer than 50% of the patients had a therapeutic response, despite genomic data that matched the actionable mutation in the patient’s tumor to a specific drug [4,5,6]. These findings highlight an important limitation at the forefront of precision medicine—that is, knowing the sequence of a tumor sample is insufficient for predicting a patient’s treatment response.

One reason for the gap between tumor mutation status and treatment response is that molecular profiling of a tumor biopsy sample is unlikely to represent the breadth of that tumor’s heterogeneity. Intra-tumoral heterogeneity, or distinct genetic and phenotypic profiles between cells within a patient’s tumor, plays a significant role in treatment resistance [7,8,9]. Genetic, epigenomic and transcriptomic changes drive intra-tumoral heterogeneity and influence how individual tumor cells respond to treatment. Heterogeneity complicates the classic precision medicine approach that relies solely on DNA mutations for targeted therapy optimization, as DNA sequencing excludes the non-genetic factors that can predict the tumor’s drug response [10].

Patient-specific tumor models, or avatars, are now providing an opportunity to assess the impact of tumor heterogeneity on cancer treatments [11]. Avatars are often cancer xenograft models, where animals are transplanted with a patient’s tumor cells and serve as “stand-ins” so that therapies can be tested and refined. The ultimate goal is for these avatars to prospectively guide the patient’s treatment plan. Traditionally, mice are used to generate patient-derived xenografts (PDX) and avatars. In these models, patient tumor cells are transplanted into immunodeficient mice. The xenografted mice are then used for the pre-clinical evaluation of drug therapies [12,13]. Mouse avatars have a high predictive capacity for their matched patients in terms of treatment sensitivity [14,15,16], but the models can take months to develop before testing can begin, which is not ideal for patients with rapidly progressing cancers [17].

Xenograft of human cancer cells into zebrafish (*Danio rerio*) has recently emerged as a powerful technique to rapidly assess tumor phenotype and drug responses in living animals at a fraction of the cost of mice [18,19]. Zebrafish PDX (zPDX) avatar models have become a clinically relevant tool for rapid, large-scale cancer therapeutic screening and can serve as a valuable complement to more commonly used avatar models. In this review, we will focus on the benefits of zPDX in assessing intra-tumor heterogeneity and accurately predicting patient outcomes in precision medicine pipelines.

## 2. Tumor Heterogeneity Complicates Precision Medicine Approaches

Inter-tumoral heterogeneity, or genetic and phenotypic differences between tumors in patients, drove the need for precision medicine in oncology. As DNA sequencing technology developed, researchers and clinicians recognized that not all tumors harbored the same mutations, even between patients with the same type of cancer. Mutations within tumors have been linked to biological behaviors and drug response, and efforts to block the effect of specific mutations gave rise to targeted therapy [20,21]. For instance, the identification of a BCR–ABL translocation in most Chronic Myeloid Leukemia (CML) patients led to the development of the selective inhibitor Imatinib and improved the 5-year survival to 90% [22,23,24,25]. In more heterogeneous cancers, navigating the tumor’s molecular profile to decide on the most appropriate treatment is challenging. For example, in breast cancer, the expression levels of the established biomarkers’ estrogen receptor (ER), progesterone receptor (PR) and HER2 were primarily used for clinical decision-making [26]. Next-generation sequencing has now revealed extreme inter-tumoral heterogeneity among breast cancer patients [27], which can be driven by diverse etiological and environmental factors. Stephens et al. found 73 unique combinations of genetic lesions in cancer-related genes across 100 breast cancer patients [28,29]. These data suggest that clinicians can assign a therapy, but whether a particular tumor’s combination of mutations would make it treatment resistant is nearly impossible to predict based on sequencing data alone.

**Figure 1:**
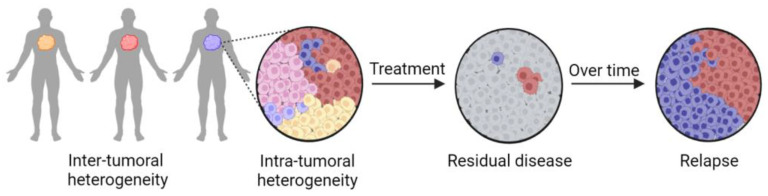
Heterogeneity in cancer takes place at multiple levels and affects patient outcomes. Inter-tumoral heterogeneity is the genetic and non-genetic differences (represented by different colors) between the same type of tumors in different patients and contributes to how patients will respond to therapy. Inter-tumoral heterogeneity is the basis for precision medicine approaches. Intra-tumoral heterogeneity is genetic and functional differences between populations or sub-clones of a single tumor (shown by different colors), identified by next-generation sequencing approaches. Following treatment of a heterogonous tumor, some sub-clones may be inherently drug-resistant or acquire alterations to provide a survival advantage, potentially resulting in patient relapse.

In addition to the heterogeneity between patients, intra-tumor heterogeneity exists within a single patient. Intra-tumoral heterogeneity means that the tumor is composed of a diverse population of cells, or sub-clones, with distinctive phenotypes, including altered genetics, epigenetics, metabolism, tumor behavior and drug responsiveness (Figure 1) [30]. Notably, the heterogeneity within a tumor is dynamic. The sub-clonal composition can change as the tumor develops and is exposed to naturally occurring cellular stressors, such as changes in blood supply, local hypoxic and anoxic conditions [31], nutrient deprivation and the associated metabolic dysregulation [32], the generation of reactive oxygen species [31], the body’s immune system [33], DNA damage [34] and outside stressors, such as drug treatment [35]. Sub-clones with alterations that impart growth advantages will survive at the expense of those with less fitness, and others will acquire new mutations that allow them to survive. Additionally, growth advantages that impact clone fitness may vary across tumor regions and locations, resulting in a complex tumor environment and architecture, which is further complicated by the introduction of therapeutic agents [36,37].

In an early study to examine the extent of intra-tumoral heterogeneity within a single tumor, Gerlinger et al. performed exome sequencing on nine spatially separated biopsy samples from a single renal-cell carcinoma patient. The analysis revealed remarkable heterogeneity, as about two-thirds of the somatic mutations were not detectable across all the samples and gene expression profiles from different regions of the same tumor displayed different prognosis signatures [38,39]. Similar intra-tumor heterogeneity has been reported in numerous cancers, including lung adenocarcinoma [40], meningioma [41], HER2-positive breast cancer [42], prostate cancer [43] pancreatic ductal adenocarcinoma [44], glioblastoma [45], colorectal cancer [46], liver cancer [47], lymphoma [48] and many others [49,50]. Tumor heterogeneity has been linked to poor patient prognosis and is regarded as one of the critical determinants of therapy resistance, treatment failure and decreased overall survival [7].

The data described above have important implications for precision medicine approaches, in that a single targeted agent is unlikely to be effective against a highly heterogeneous tumor. Sequencing of multiple, spatially separated biopsies may be required to reveal the degree of heterogeneity within the tumor and to assign the correct targeted drug panels to the patient. At the same time, a tumor’s drug responsiveness is often a combination of genetic and non-genetic factors that DNA sequencing may not fully encompass. For precision medicine to reach its full potential, there is a need to define the functional impact of intra-tumoral heterogeneity on drug response and the behavior of individual sub-clones. By combining tumor-specific models and drug testing with sequencing data, a patient’s treatment plan could be tailored based on the sensitivity of each sub-clone to targeted therapeutics.

## 3. Current Patient-Derived Avatar Models Are Not Suitable for Clinical Timelines

A patient-derived avatar model, in which a patient’s tumor is transplanted into an animal or grown as a 3D organoid culture, serves as a functional surrogate for that patient. Avatars provide an important venue to interrogate tumor behavior and drug responsiveness while sparing the patient from undesirable side effects and wasted time of ineffective therapies. Ultimately, findings from avatars can be translated back into the same patient’s treatment plan and can also inform the decision-making for a larger group of patients with tumors with matched genetic or molecular profiles [13,51,52].

Currently, mouse patient-derived xenografts (mPDX) are regarded as the most powerful model for predicting clinical outcomes. Several studies have demonstrated a high fidelity between patient samples and mPDX in histology, genomic and transcriptomic profiles [53,54]. In simple terms, a patient’s sample is implanted in immunodeficient mice, and mice are treated with drug combinations that patients would receive for that cancer type. Mouse avatars have a reasonable correlation with patient outcomes. For example, in metastatic sarcoma patients, 13 out of 16 (81%) were reported to have similar clinical responses to their avatars [55]. Patient-derived organoids (PDO) are also used as avatar models. They are self-organized, 3D cell cultures established from the patient’s biopsy and maintained in vitro. PDOs enable the examination of cell-to-cell interactions and provide insight into the effect of the tumor microenvironment on the tumor cell behavior [56]. PDO avatars can also forecast patient response to chemotherapy. For example, PDOs provided a high predictive value of 88% in metastatic gastrointestinal cancer [57].

These models have been used for several years but have significant limitations. First, for unknown reasons, not all patient samples will engraft into mice to form a mouse PDX, and some patient samples will not grow as organoids. For example, the success rate for generating mPDX of breast and renal cancers was 13% and 8.9%, respectively [19]. Similarly, for patient-derived organoids, a low success rate of 16% is reported in advanced prostate cancer [58]. The unpredictable engraftment success of these avatar models limits their utility to a random subset of individuals, with certain patient tumors unable to survive in these conditions.

A critical limiting factor of these models is that the time they take to develop is incompatible with the rapid pace with which decision-making must occur in the oncology clinic [59]. In clinical practice in the United States, the median time between diagnosis and initiation of therapy is 26 days for colon cancer to as long as 79 days for prostate cancer [60]. Timelines to develop mPDX avatars are measured in months, with the average mPDX requiring 6–8 months to develop [11]. Some mPDX models have an even longer latency. Prostate cancer mPDX may take up to 37 months to engraft and become established in mice [61]. This timeline is not feasible in particularly aggressive cancers, such as certain glioblastomas, where patients progress and can succumb to their disease within weeks [62]. Although PDOs are cultured in vitro and have shorter generation timelines, they still require 4–6 weeks to develop from patient tissue samples. Because of this extended timeline, the current cancer avatar models are generated in case the patient develops relapse or to inform treatment plans of newly diagnosed patients with tumors with similar molecular profiles. The promise of cancer avatars, which is that there will be a patient-specific tumor model to help guide clinical decisions from diagnosis onwards, has not been fully realized with the avatar models that are currently available.

## 4. Zebrafish Patient-Derived Xenografts (zPDX) Fill Gaps in Current Model Systems

Use of zebrafish as cancer avatars can overcome some of the limitations of the current model systems. Human cancer cells can be engrafted into zebrafish larvae less than five days old or adult zebrafish more than 30 days old [63,64]. Procedures to generate patient-derived xenografts (zPDX) in both the larvae [65] and adult zebrafish [66,67] are well-established, and each has its unique benefits. zPDX into larvae are ideal for large-scale drug screening, as hundreds of animals can be easily xenografted relatively quickly. Larval zebrafish are well-established as a pharmacological tool, as they can live in 96-well plates and readily absorb compounds from the water [68]. The immune system in zebrafish is not fully established until 21 days post-fertilization [69], and drug testing is usually completed within 96 h after xenograft. Immune rejection is typically not a concern in larval zPDX. For xenograft into adult fish, animals must first be made immune-deficient through gamma irradiation [70] or immunosuppressant treatments by dexamethasone [71]. The zebrafish immune system typically regenerates within three weeks, and the xenografted tumor will eventually be rejected. The zPDX in this adult model is, therefore, time-sensitive. More recently, Moore et al. reported a genetically modified immunodeficient zebrafish with mutations in recombination activating gene 2 (*rag2*), DNA-dependent protein kinase (*prkdc*) and janus kinase 3 (*jak3*), resulting in the loss of T, B and natural killer cells [72]. *prkdc*^−/−^, *il2rga*^−/−^ immune deficient fish were shown to robustly engraft a wide array of human cancers at 28 days. Importantly, the phenotypes of the human xenografted cells in zebrafish resembled that of mouse PDX [64], indicating that key properties of human cells were retained whether they were transplanted in mammals or fish. Use of adult fish also permits oral gavage or intraperitoneal injection of drug treatments. Although this limits the model to more modest numbers in drug testing, the delivery method serves as an accurate and relevant mimic of patient dosing and could provide useful insight in precision medicine approaches [66].

**Figure 2:**
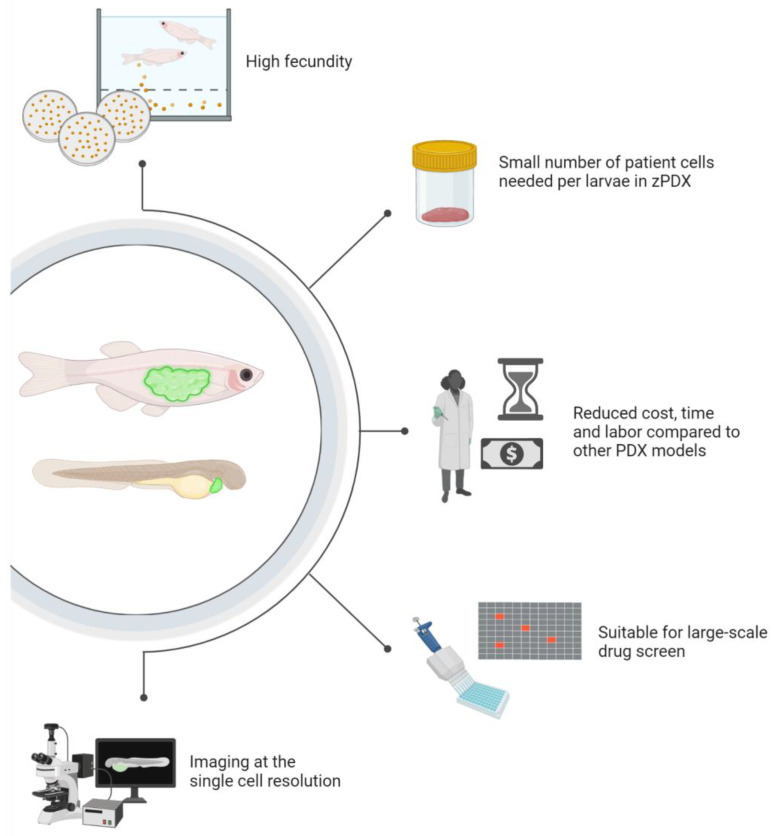
Unique features of the zebrafish make them powerful candidates for use as cancer patient avatars. From the top, clockwise: Zebrafish are highly fecund, with a mating pair generating hundreds of offspring per week for use in experiments. Larval zPDX can be generated with just hundreds of cells when patient material is limited. The low cost, labor and time required to generate zPDX, coupled with the available automation techniques, make zebrafish useful for drug screens to identify effective candidate treatments among many available therapeutics. Finally, the optical clarity of the zebrafish *Casper* stain makes them excellent for high-resolution imaging down to a single-cell level.

There are several benefits of zPDX (Figure 2) that make this model a valuable complement to mouse and organoid avatar systems. The most important is the time needed to generate the xenograft. Cells can be visualized immediately after transplant in a larval zPDX, and in most cases, drug treatments start 24–48 h post-transplant. Workflows are typically completed within a week [73,74]. Adult immunodeficient fish require 7–28 days for robust engraftment [64], significantly less time required than the 3–8 months typically needed for mouse PDX engraftment or 6–8 weeks for in vitro organoid formation. Zebrafish avatars can, therefore, more readily fit into clinical decision-making timelines for cancer patients.

Zebrafish are also low-cost and accessible to a wide range of laboratories. The use of mouse models requires a substantial investment in infrastructure for mouse housing and separate facilities for imaging and procedures. The cost of care and housing for four mice averages $1.05 per day, and a breeding pair will generate ~6–8 pups after 21 days of gestation. In contrast, a tank of a dozen zebrafish can be maintained for $0.25 per day [75], and each mating pair of zebrafish produces >100 eggs weekly. Facilities housing even as few as 50 animals could complete an extensive number of larval zPDX and drug testing experiments weekly in 96-well plates [76,77]. While mouse PDX will continue to play an essential role in testing small numbers of drugs in mammalian systems, their cost and labor-intensive procedures limit their applicability in large-scale screens to test the efficacy of precision medicine compounds. Zebrafish models can fill this gap.

In efforts to reduce the labor surrounding the use of zPDX and potentially increase the throughput of drug screening, steps in developing zebrafish research models have been automated (Table 1). For instance, a robotic injection system can inject up to 2000 embryos per hour with a success rate of 99% for high-throughput assays [78]. This method has been reported for injecting DNA to obtain transgenic animals. Nonetheless, there is potential for the extension of this work for large-scale xenografting in embryos. Imaging steps can also be automated with several approaches. For instance, the VAST Bio-Imager can image up to 100 larvae per hour with an 85% imaging success rate [79]. Several groups have automated image acquisition and processing for large-scale cancer cell counting [80], evaluation of tumor dissemination [80] and generation of tumor migration data [81].

Finally, zPDX provides intravital imaging approaches that are not possible in mice, which can provide new information on tumor phenotypes and drug response. Zebrafish larvae are optically transparent, and xenografted cells are typically stained with a fluorescent dye or express fluorescent proteins, allowing cells to be monitored in living animals from the moment of xenograft, through drug treatment and onwards [82]. For example, Mercatali et al. described a zPDX model of breast cancer bone metastasis in which labeled cells were observed to extravasate and colonize the caudal hematopoietic tissues (CHT), a bone marrow-like niche in zebrafish [83]. Similarly, Asokan et al. used selective plane illumination microscopy (SPIM) to record the real-time migration of cancer cells. This method was able to differentiate the dissemination patterns and speed of dissemination between breast cancer and leukemia cells [81]. Almstedt et al. integrated live fluorescent imaging and artificial intelligence-driven image analysis for a real-time evaluation of GFP-tagged glioblastoma orthotropic xenografts, examining growth, invasion and survival [84]. In adult models, Yan et al. engrafted human rhabdomyosarcoma cells into the peri-ocular muscle of immune-deficient *prkdc*^−/−^, *il2rga*^−/−^ zebrafish. This site is accessible to imaging approaches to allow for the dynamic visualization of the pharmacodynamics of drug treatment at a single-cell resolution. In this experiment, before engraftment, human rhabdomyosarcoma cells were made to stably express the FUCCI4 cell-cycle fluorescent reporter. Seven days after engraftment, single and combination drug treatments were administered by oral gavage. Olaparib and temozolomide combination therapy resulted in a higher number of G2-arrested cells compared to control animals [64]. This combination is now in phase I clinical trial in pediatric rhabdomyosarcoma patients (clinicaltrials.gov identifier: NCT01858168) [85].

The unique benefits of the zebrafish, particularly the short time required to generate the zPDX and the scale at which drug screening can be completed, make them an ideal tool for cancer avatar models in clinical diagnostic pipelines. Imaging approaches, alone or in combination with drug treatment, can also provide a means to rapidly assess the sub-clonal phenotypes within a heterogenous tumor that could significantly impact therapy response. This latter attribute is currently not possible in mPDX.

**Table 1:** Overview of automation approaches with potential application in zPDX.

Automated Step	Equipment and Software Involved	Application	Speed or Efficacy	Accuracy	Limitations	Ref.
Imaging and Image analysis	Automated microscopy platform, IncuCyte S3 and CNN for image analysis	Characterize the tumor growth in the xenografts in vivo	One image every 4–6 h over 4 days	77% agreement with manual analysis	Difficulty of identification of early signs of death	[84]
Image analysis	Faster R-CNN algorithm, Inception Reset V2 feature extractor	Counting of tumor cells in zPDX	Not mentioned	85% average precision	Larger datasets are considered for higher accuracy	[86]
Image analysis	SPIM for imaging, CellProfiler, CellTracker and R open source tools	Real time visualization and tracking of metastatic cancer cells in zPDX	Not mentioned	Differentiate the dissemination patterns between two types of cancers	Not mentioned	[81]
Imaging	VAST BioImager System, upright microscope, LP Sampler	Automate zebrafish larva handling, positioning, orientation and imaging	100 and 75 larvae/h sampling from bulk and 96-well plate	86% and 97% success rate imaging from bulk and 96-well plate	Applicable for 2–7 dpf larva, high storage space for obtained images	[79]
Imaging and treatment administration	Automated pipetting workstation, automated imaging system (ImagXpressMICRO)	Live imaging of zPDXand automated application of therapeutics	Not mentioned	Not mentioned	Not mentioned	[87]
Imaging and treatment administration	ImageXpress Micro System, JANUS automated workstation	Collect High-Content images for adult and embryo zPDX	Not mentioned	Not mentioned	Not mentioned	[88]
Injection	Motorized stage with paired controller, motorized micro-manipulator, “injectman II”, z-calibration unit	Semi-automate injection	2000 embryos/h	99% success rate	Automation is reported for injection into embryos and requires commercial injection needles	[78]
Injection and screening	Automated microinjection system and COPAS	Automate injection into zebrafish embryos and fluorescence quantification	3000 embryos/15 min for sorting step	Not mentioned	Not mentioned	[89]
Imaging, image pre-processing and analysis	CLSM platform with movable stage, Image-Pro Software	Quantification of human cancer dissemination in zPDX	Less than 5 min per plate for imaging	Dissemination in zPDX significantly correlated with metastatic behavior in mouse model	Confocal microscopy is required	[80]

CLSM: Confocal Laser Scanning Microscope, CNN: Convolutional Neural Network, COPAS: Complex Object Parametric Analysis and Sorting, dpf: days post fertilization, LP: Large Particle, R-CNN: Regions with Convolutional, Neural Network, SPIM: Selective Plane Illumination Microscopy, VAST: Vertebrate Automated Screening Technology, zPDX: Zebrafish patient-derived xenografts.

## 5. Zebrafish Are Clinically Relevant Cancer Avatars Models

Xenograft of human cancer cell lines into zebrafish is now a well-established technique. Researchers have found that important behavioral properties of cancer cells, such as proliferation, migration, matrix remodeling and angiogenesis, among others, can be observed in zebrafish xenografts, despite zebrafish being highly dissimilar to humans. For example, in 2006, in the first human cancer cell xenograft, melanoma cells were observed to proliferate, migrate and form masses that participated in angiogenesis [90]. Nicoli et al. similarly used human cancer cell line xenografts in zebrafish to mimic the initial stages of tumor angiogenesis. The model was able to discriminate between two endometrial cancer lines that differ for VEGF and FGF expression and angiogenic behavior. In agreement with data from nude mice, angiogenesis was observed in 75% of zebrafish larvae xenografted with the highly angiogenic line and none from the poorly angiogenic line. Treatment with anti-angiogenic compounds used in mammals diminished neovascularization in all zebrafish larvae without affecting physiological vessel development [91]. This early data proved that zebrafish xenografts can provide useful information about human cancer cell phenotypes and that anti-cancer compounds can be relevant in a zebrafish xenograft setting.

Following the success of xenograft of human cancer cell lines, patient-derived xenografts models in zebrafish have been developed and used to assess different attributes of tumor behavior. zPDX models have since been established in multiple cancer types, including gastric cancer [92], colorectal cancer [73], breast cancer [83], non-small cell lung adenocarcinoma [93], pancreatic ductal adenocarcinoma [94] and others [68,74,95]. Ai et al. highlighted the importance of an in vivo system to patient-derived cells by comparing the transcriptomic profile of glioblastoma multiforme (GBM) cells that were xenografted to zebrafish larvae brain to GBM cells grown in vitro in typical cell culture conditions. The authors used principal component analysis (PCA) to show that in vitro grown cells clustered together and away from the xenografted cells. The xenografted cells showed significant gene enrichment in categories, such as tumor metastasis, cell migration and epithelial-to-mesenchymal transition. This transcription identity suggests that the zPDX models can recapitulate the supportive tumor microenvironment that patient-derived cells need [95].

Zebrafish PDX has also been compared to mouse PDX models for use as cancer avatars. Ali et al. developed a combined mouse and zPDX testing platform by engrafting 39 preserved mPDX tissue fragments of non-small cell lung cancer into zebrafish embryos. The zPDX accurately predicted lymph node involvement with 91% sensitivity. In addition, the response of zPDX to standard treatments correlated very well with the response in mPDX and patients [93]. Fior et al. directly compared the chemosensitivity profile between colorectal mice and zebrafish PDX. In both models, chemotherapies significantly increased apoptosis and reduced proliferation. The differential sensitivities to chemotherapy in some colorectal cancer cells uncovered by zPDX models were later validated in mPDX models [73].

While the above data support a role for zPDX in the avatar setting, there are some limitations to consider. Cell-signaling between cells within the tumor microenvironment can profoundly impact xenograft behavior and drug response. Cell-to-cell communication can occur between zebrafish and human cells to impact xenograft behavior. For example, in a triple-negative breast cancer xenograft model, the zebrafish cytokine Cxcl12 was found to trigger the human chemokine receptor CXCR4 on engrafted cells, which was suggested to drive the formation of breast cancer micro-metastases in the zebrafish [96]. However, only 70% of genes are conserved between the zebrafish and the human genome [97], so some receptor/ligand interactions and signaling pathways in mammalian tissue might not be present in the zebrafish tumor microenvironment. For instance, mouse or human growth factors cannot stimulate hematopoietic precursors in zebrafish [98]. Recently, editing the zebrafish genome was employed to produce a more accurate tumor microenvironment for human xenografts. Rajan et al. described the generation of a transgenic “humanized” zebrafish that expresses human-specific cytokines, resulting in enhanced human leukemia cell migration and proliferation after xenograft transplantation [99].

Another possible issue in the generation of zebrafish cancer avatars is the difference in temperature between the xenograft and the host. Zebrafish are housed at 28.5 °C, and the physiologic temperature for human cells is 37 °C. Most larval zPDX studies house xenografted animals at 34 °C. When developing larvae are incubated at a higher temperature, they may experience changes in metabolic, immune and inflammatory responses and can develop malformations that affect the integrity of the host system [100,101]. Incubating the human tumor cells at a lower temperature than 37 °C will affect their proliferation rate [102]. This limitation can partially be resolved using the adult *prkdc*^−/−^, *il2rga*^−/−^ immunodeficient fish, which are adaptable to surviving at 37 °C for more extended periods [64].

A final limitation of zebrafish in the avatar setting is that they are not ideal for orthotopic transplants. In this model, cancer cells are xenografted into an animal’s organ or tissue that matches the tumor’s histotype. Orthotopic xenografts often provide an ideal microenvironment to recapitulate tumor behavior, phenotypes and drug responses. For example, in zPDX of human glioblastoma, cells were found to proliferate and invade when transplanted into the zebrafish brain, the orthotopic site, but remain static when xenografted into the zebrafish yolk sac [103,104]. Due to the absence of some organs in zebrafish, such as the lungs, and the small size of other organs, orthotropic engraftment might be challenging for certain cancers [102]. Table 2 provides a summary on the current status, advantages and disadvantages of each model.

## 6. Zebrafish Bring Pharmacology Strengths into Precision Medicine Pipelines

Zebrafish have been used for many years in pharmaceutical research in large-scale, in vivo small-molecule screens [105]. Their pharmacology success is one of their significant benefits in the cancer avatar field. Because of this history, drugs are now established to have conserved effects between humans and zebrafish. For instance, research showed that human cardiotoxic drugs induced pericardial edema and circulatory disturbances in zebrafish, compared to no signs of cardiovascular toxicity for non-cardiotoxic drugs, with a 100% prediction success rate [106]. Zebrafish are a well-established model for pharmacokinetics, with many phases I and II metabolic reactions used in drug processing conserved between zebrafish and mammals [107,108,109]. Zebrafish also have direct orthologs to human cytochrome P450 (CYP) enzymes, which metabolize hepatically cleared drugs. The conservation of metabolic processes between zebrafish and humans indicates that drugs are likely processed and utilized by zebrafish and human patients in much the same way [110].

Drug screens in zebrafish can directly impact human disease, as chemical screens in zebrafish have identified several drugs across a wide range of indications that are now in different phases of clinical testing [111,112]. For example, a screen in zebrafish led to the identification of Dorsomorphin, the first bone morphogenetic protein (BMP) inhibitor. Derivatives of this compound are being clinically tested for fibrodysplasia ossificans progressiva and anemia of inflammation [113]. Pharmaceutical companies are also realizing the value of the zebrafish model and have established in-house zebrafish facilities or are collaborating with external zebrafish Contract Research Organizations (CROs) [114].

The success of zebrafish models in the drug discovery field and the translatability of their findings to human disease makes them robust and reliable candidates for drug sensitivity profiling along the precision medicine pipeline. Larval zPDX provides a whole-animal venue for evaluating drug pharmacokinetics, pharmacodynamics and toxicity. At the same time, these xenografted animals are easily manipulated in a 96-well plate [105]. The skin of zebrafish larvae has a high permeability to enable drug screening by immersion, making the drug testing process for xenografts much faster than injecting mice [115]. However, this method presents a challenge for drugs with limited water solubility. Drug treatment at temperatures lower than 37 °C might also influence the efficacy of certain chemotherapeutic agents [102]. Finally, drug screens and other tumor characterization assays are typically restricted to 7–8 days post-engraftment in larval zPDX before the immune system begins to reject the transplant [116]. The window for successful engraftment will limit the drug exposure time and might influence the drug response. This short time frame means that drugs that alter cellular proliferation or induce apoptosis are most likely to strongly impact the xenografted cells. Drugs that take a longer time to result in cell death through mechanisms involving interference with the cell cycle or epigenetic alterations, for example, may not be able to demonstrate a significant effect in this short-term model [117].

As zebrafish cancer avatars become a more established tool, further efforts will be needed to understand the pharmacokinetics and the related ADME (absorption, distribution, metabolism and excretion) factors of therapies in zPDX. This information is necessary to determine whether a targeted therapy that failed in a zPDX was ineffective because the tumor was not responsive or if the animal model itself caused an issue. For example, the drug may not be absorbed well into the fish, metabolized correctly or able to reach the cancer cells. Building these datasets for zPDX on common precision cancer medicines will allow zebrafish to become a reliable avatar model and a strong complement to mouse PDX.

## 7. Tank to Bedside: Zebrafish Avatars for the Prediction of Patient Outcomes

Several studies evaluated the ability of zPDX to predict patient responses (Table 3). A subset of these utilized a co-clinical trial design to implement a zebrafish avatar model [94,118,119,120]. The concept of co-clinical trials arose from the increased understating of inter-tumoral heterogeneity, or the individuality of each patient’s disease, and the need to optimize therapy at the patient level. A co-clinical trial aims to enable efficient patient treatment while minimizing the unwanted administration of ineffective therapies, which could have undesirable side effects. A parallel arm is added to the standard clinical trial design using PDX avatars to re-capitulate the patient tumor and provide a venue for testing and screening for optimal therapy [17]. Zebrafish avatars have shown promise in these types of studies. Di Franco et al. transplanted patient-derived pancreatic cancer cells into the zebrafish embryos’ yolk to screen the treatment plan chemotherapy agents. After two days of treatment, the response to treatment was evaluated by comparing the relative tumor area to the control. A statistically significant reduction in relative tumor area was reported in 6 of the 15 patient samples. Using a conversion factor to compare the chemotherapy efficacy reported in the literature as a percent partial response reported in humans, the group demonstrated that chemotherapy in zPDX reflected the efficacy of chemotherapy in clinical settings [118].

The result of this work formed the basis of the first zebrafish co-clinical trial XenoZ (clinicaltrials.gov identifier: NCT03668418), an observational, prospective clinical trial on patients with pancreatic and gastrointestinal cancers. The study will recruit 120 patients to demonstrate the zPDX model’s efficacy in predicting the optimal treatment regimen [121]. The authors first reported a conversion factor for determining the equivalent dose of chemotherapy in zebrafish; this factor was determined by combining data from zebrafish safety studies (maximum tolerated dose) and efficacy (significant decrease in tumor area). Twenty-four patients with pancreatic, colon and gastric cancers undergoing chemotherapy were enrolled in the study, and their biopsies were used to generate avatar models. Avatars were tested for chemotherapy response and evaluated using a zebrafish-adapted form of the Response Evaluation Criteria In Solid Tumors (RECIST) based on the change in tumor area in the zPDX avatar. The sensitivity of different engrafted tumor types to administered chemotherapy was consistent with that reported in the clinical context. For example, in colon cancer avatars, combination treatment (FOLFOX, FOLFIRI and FOLFOXIRI) were more effective than a single agent 5-FU [120], just as it was in patients.

zPDX avatars could also predict treatment response in pancreatic ductal adenocarcinoma (PDAC) [94]. In this study, patients were considered non-responders if their cancer reoccurred within one year and were responders if they remained disease-free. In the zPDX avatar model, responders were defined as having more than a 50% decrease in tumor size in response to treatment. Out of the 31 patients who provided samples, 16 were followed up for a median time of 19.3 months. The zPDX avatars indicated that 7 out of the 16 patients were responders, and 9 were non-responders to treatment. Of those, only 1 patient out of the 7 (14.3% error) zPDX responders reported a cancer recurrence, while 6 of 9 (66.7% accuracy) patients that the zPDX avatars identified as non-responders had a cancer recurrence. The zPDX models could, therefore, accurately identify patient response to chemotherapeutic agents.

In addition, and under the same co-clinical trial, a zPDX avatar model was developed with samples from patients with colorectal cancer and used the adaptive RECIST criteria to predict chemotherapy response when treated with 5-FU, FOLFIRI, FOLFOX, FOLFOXIRI for two days. These zPDX avatars predicted chemotherapy response with 75% accuracy [119]. A similar trial is planned, called the Evaluation of Tumor Growth and Oncological Treatment in Patients With Colorectal Liver Metastases (CRLM) Using Zebra Fish Embryo Model (CRLM-Z) (clinicaltrials.gov identifier: NCT05289076). This trial is an observational prospective cohort study set to enroll 40 patients. Tumor response to different combinations of therapies will be tested in patients and zPDX, and based on the success of the observational study, interventional studies may be planned [122]. zPDX avatars now serve as a powerful translational tool for precision medicine and may have the potential to support clinicians in making individualized and informed treatment decisions for their patients [49,117,123].

**Table 3:** Studies correlating zebrafish PDX outcomes to patient outcomes in different cancers.

Cancer	Zebrafish Age	Number of Patient Samples	Outcome Assessed	Therapeutic Regimen	Xenograft Characterization	Criteria for Zebrafish Response Evaluation	Criteria for Human Response Evaluation	Concordance ^1^	Ref
CRC	48 hpf	36	Chemotherapy response	5-FU, FOLFOX, FOLFIRI and FOLFOXIRI	Engraftment success and change in equivalent tumor volume (%∆V)	Adaptive RECIST ^2^ Criteria	RECIST criteria	75%	[119]
	48 hpf	2	Radiotherapy sensitivity	25 Gy + 5-FU	Engraftment success, Tumor size and Apoptosis of tumor cells	Tumor size and cell death	MRI response and rectosigmoidoscopy	100%	[124]
	48 hpf	5	Chemotherapy response	FOLFIRI, FOLFOX and Cetumixab	Engraftment success and tumor size	Tumor size, angiogenesis potential, apoptosis and metastatic potential	Disease recurrence, CEA levels and treatment response	80%	[73]
NSCLC	48 hpf	4	Chemotherapy response	Erlotinib and Paclitaxel	Engraftment success and tumor size	Relative tumor sizes, dissemination	Not mentioned	Not mentioned	[93]
	48 hpf	21	Chemotherapy response	Osimertinib, Pemetrexed, Docetaxel and Cisplatin	Engraftment success and tumor size	Tumor size	RECIST criteria	76.9%	[125]
PDAC	48 hpf	31	Chemotherapy response	Gemcitabine, GEMOX, GEM/nab-P and FOLFOXIRI	Engraftment success and change in equivalent tumor volume (%∆V)	Adaptive WHO Criteria ^3^	Adjuvant treatment administration, disease recurrence rate and DFS	66.7% for cancer recurrence	[94]
	48 hpf	15	Chemotherapy response	Gemcitabine, GEMOX, GEM/nab-P and FOLFOXIR	Engraftment success, Larvae survival rate, Histology and Immunohistochemistry	Mean RTA	PFS, overall health and failure-free survival	Not mentioned	[118]
TNBC and CRC ^4^	48 hpf	2	Bevacizumab response	Bevacizumab	Engraftment success	Apoptosis and metastatic potential	Bevacizumab treatment resistance	Resistance predication ^5^	[126]
Pancreatic CRC and GC	48 hpf	24	Chemotherapy response	5-FU, FOLFOX, FOLFIRI, FOLFOXIRI, GEM, GEMOX, GEM/nab-P, FLOT and ECF	Engraftment success	Tumor size, Adaptive RECIST ^2^ criteria	Not mentioned	Not mentioned	[120]
GC	48 hpf	14	Chemotherapy response and dissemination pattern	5-FU, docetaxel and apatinib	Engraftment success and larvae survival rate	Cell proliferation rate	Clinical and histopathological characteristics	Not mentioned ^6^	[92]

^1^: between patient outcome and zebrafish PDX outcome, ^2^: Progressive Disease (PD): increase of ≥20% in the relative stained area at 2 dpi/2 hpi., Stable Disease (SD): decrease of <30% or increase of <20% in the relative stained area, at 2 dpi/2 hpi., Partial Response (PR): decrease of ≥30% but <90% in the relative stained area at 2 dpi/2 hpi. Complete Response (CR): decrease ≥90% in the relative stained area at 2 dpi/2 hpi. ^3^: Progressive Disease (PD): Increase ≥ 25% in the relative stained area at 2 dpi, Stable Disease (SD): Decrease or increase <25% in the relative stained area at 2 dpi, Minor Response (MR): Decrease ≥25% but <50% in the relative stained area at 2 dpi, Partial Response (PR): Decrease ≥50% but <90% in the relative stained area at 2 dpi, Complete Response (CR): Decrease ≥90% in the relative stained area at 2 dpi, ^4^: targeting VEGF signaling, ^5^: Bevacizumab resistance in zebrafish xenografts seem to predict resistance in matched human case studies, ^6^: Patient clinical information could not be tracked, Chemotherapy protocols: 5-FU: 5-Fluorouracil, ECF: 5-Fluorouracil + Cisplatin + Epirubicin, FLOT: 5-Fluorouracil + Lederfolin + Oxaliplatin + Docetaxel, FOLFOX: 5-Fluorouracil + Lederfolin + Oxaliplatin, FOLFIRI: 5-Fluorouracil + Lederfolin + Irinotecan, FOLFOXIRI: 5-Fluorouracil + Folinic acid + Oxaliplatin + Irinotecan, GEM: Gemcitabine, GEMOX: Gemcitabine + Oxaliplatin, GEM/nab-P: Gemcitabine + nab-Paclitaxel, CRC: Colorectal cancer, DFS: Disease-free survival, GC: Gastric cancer, Gy: Gray, NSCLC: Non-Small Cell Lung Cancer, PDAC: Pancreatic ductal adenocarcinoma, PFS: Progression-free survival, RECIST: Response Evaluation Criteria in Solid Tumors, RTA: Relative tumor area, TNBC: Triple-Negative Breast Cancer.

## 8. Zebrafish Avatars Provide New Opportunities to Assess the Impact of Intra-Tumoral Heterogeneity in Precision Medicine

Mouse, organoid and zebrafish cancer avatars have been used successfully in patient-specific drug testing. Although intra-tumoral heterogeneity has not yet been directly assessed in avatar models, heterogeneity plays a significant role in whether the avatar and the patient respond to the treatment. Genetic and non-genetic factors, such as changes in the epigenome, transcriptome and metabolome, can impact how each sub-clone within a tumor responds to therapy. Every sub-clone in a patient’s tumor must be eliminated for the patient to have a good outcome with no chance of later relapse. While genomic and molecular sequencing of the tumor can provide insight into actionable mutations and provide leads on effective precision medicine, avatars fulfill the need to functionally assess how intra-tumoral heterogeneity impacts therapy response. Ultimately, avatars can take some of the guesswork out of patient treatment. If specific therapies do not eliminate cancer in the avatar model, other drugs can be tried until all sub-clones respond. Using avatars in clinical decision-making pipelines can help predict what drug combinations might be successful in a heterogenous tumor while sparing patients the side effects and wasted time of ineffective therapies.

Mouse models are the most widely used system for pre-clinical testing of personalized medicine strategies [127,128]. However, some data suggests that current methods of generating mouse PDX may not be ideal for recapitulating patient heterogeneity in avatar models. Generating a stable mPDX for drug testing generally requires serial transplanting human tumors through three or more passages in mice [129]. mPDX generated after this degree of serial transplantation exhibits more aggressive tumor phenotypes in terms of proliferation rate, metastasis and invasion. Xenograft passage number in mice was associated with increased tumor growth and histopathological features of higher tumor grade [130]. Genomic alterations caused or selected by passaging also affected the sensitivity of tumor cells to drugs, especially at later passages [131,132].

In addition, clonal dynamics can change with serial transplantation of mPDX. Belderbos et al. examined the clonal behavior among leukemia mPDX models. Primary recipients had a diverse clonal composition, but each serial transplantation of leukemia resulted in a 30–90% reduction in the number of sub-clones [133]. Serial transplantation of breast cancer mPDX also caused changes in growth kinetics across multiple samples [134]. These findings suggest that the development of mPDX models and mouse cancer avatars may be impacted by the selective pressure on xenografted cells to adapt to the murine environment. A heterogeneous tumor in a patient is dynamic and responsive to stressors, so the fittest clones survive to drive tumor growth. However, the microenvironment in the patient that drives this selective pressure is very different from the selection that occurs during the months required for human tumor cells to be engrafted and serially passaged through mPDX. These methods may result in the potential infidelity of the model as it is related to the heterogeneity of the primary patient sample [135]. Therefore, drug screening in these avatars may not accurately represent tumor response in patients.

Larval zebrafish PDX could address this issue. While the human cancer cells would still need to survive in a foreign tumor microenvironment, cells are xenografted into larvae directly from the primary tumor after dissociation into a single-cell suspension [68,83,92,94,95,119]. Experiments are completed within 5–10 days. This short time frame gives the larval zPDX model the potential to better preserve the heterogeneity of the patient’s tumor. Xenografts in adult immunocompromised zebrafish can persist for a longer time and have been assessed up to 60 days post-transplant. Like mouse PDX, adult zPDX might be selective for specific clones capable of adapting to a zebrafish microenvironment. Overall, further investigation is needed to elucidate clonal behavior in both larval and adult zPDX, as this model becomes more commonly used as cancer avatars.

**Figure 3:**
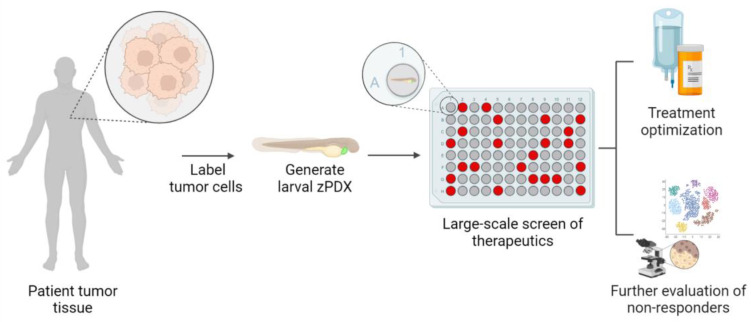
Larval zPDX avatars can provide methods of treatment optimization and uncovering mechanisms of therapy resistance. Larval zebrafish can be xenografted with a few hundred patient tumor cells per animal, effectively breaking apart intra-tumoral heterogeneity. Larval zPDX are treated in 96-well plates to assess drug sensitivities, which could help inform treatment plans. Cells from non-responding zPDX can be isolated and further characterized via genomic, transcriptomic and histopathologic tools. These data can help optimize more effective therapies for the patient.

Zebrafish also provide opportunities to dissect intra-tumoral heterogeneity in ways that are not possible in mouse models. Heterogeneity within a tumor arises from the cellular subgroups with different genomic and phenotypic characteristics, which can ultimately result in therapeutic resistance and treatment failure [136]. Larval zPDX provides an opportunity to break apart heterogeneity into individual animals, in that 100–800 patient cells can be transplanted per animal, and hundreds of larvae can be transplanted with a patient’s tumor [92]. The heterogeneous tumor would now be subdivided into smaller parts, and these zPDX avatars can rapidly test therapeutic regimens and evaluate treatment responses. Some zPDX may completely respond to therapy, and cancer cells will be eliminated. Other zPDX may not respond, depending on which sub-clones are in the xenograft (Figure 3). Based on individual drug responses, combinations of therapies could be designed to be the most effective at eliminating all sub-clones within a heterogeneous tumor. Since tumor cells will be labeled before xenograft with a fluorescent dye, the resistant clones can be harvested and analyzed using sequencing approaches to uncover mechanisms of resistance that could inform second rounds of drug testing. This type of assay has the potential to provide a snapshot of the patient’s tumor landscape in days [65], compared to mouse PDX models that require months [17,137]. The large number of animals that can be involved in these studies provides statistical power, and the single-cell resolution that zebrafish larvae provide in imaging approaches may allow for the identification of previously undetectable drug-resistant sub-clones [66,73,138].

**Figure 4:**
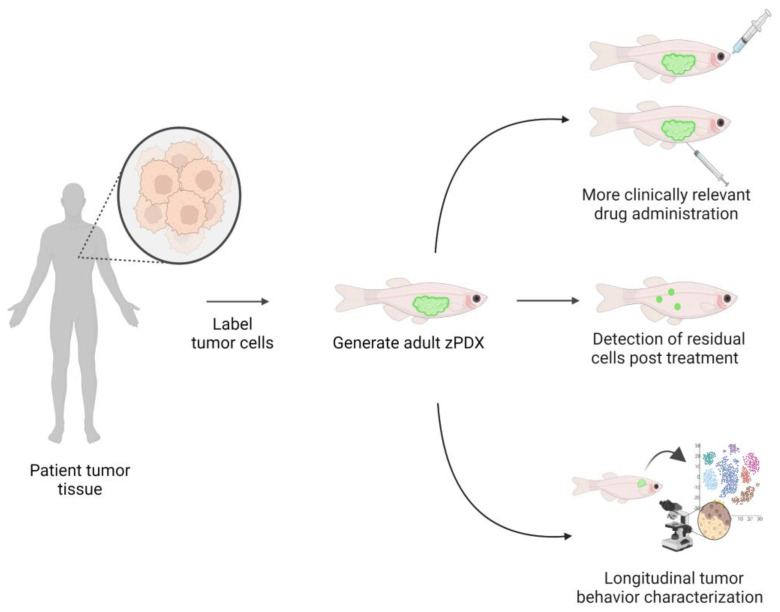
Adult zPDX avatars provide a longitudinal analysis of heterogeneous tumors. *Casper*-strain *prkdc*^−/−^, *il2rga*^−/−^ immune-deficient zebrafish PDX can be engrafted with up to 10^6^ cells, mimicking the patient’s intra-tumoral heterogeneity in a single animal. Adult zPDX provides a clinically relevant route of drug administration, such as oral gavage and intraperitoneal injection. The optical clarity of the *Casper* zebrafish enables the detection of residual disease and analysis of relapse phenotypes. Adult zPDX can maintain engraftment for more than 28 days, which enables extensive evaluation of tumor behavior and therapy response, histological and gene expression analyses reflect the clonal composition of remining cancer cells (represented in the data plot by different colors).

As a complement to larval zPDX, an immunodeficient *Casper* strain *prkdc*^−/−^, *il2rga*^−/−^ zebrafish PDX model will allow more time for longitudinal evaluation of sub-clonal behavior, as engraftment is established for more than 28 days and up to 60 days. These animals require a transplant of 10^5^–10^6^ cells per fish, so each animal will have a heterogenous tumor that mimics the patient’s sample [64]. The optical clarity of the pigment-free *Casper* zebrafish would allow for the characterization of tumor phenotypes pre-treatment and during treatment in live animals. Non-responsive cells can also be quickly assessed and harvested for tumor profiling. In particular, this model could be helpful in identifying clones that drive relapse formation, potentially revealing individualized information about the relapsed tumor’s genetic and molecular profiles that would support later treatment decisions (Figure 4).

## 9. Conclusions

The reduced timeframe for the generation of zPDX avatars, their large-scale potential in drug screening and reduced use cost compared to mouse models are all features that make zPDX an increasingly popular and attractive model for precision cancer medicine (Figure 2). Larval zPDX is now proven to be a clinically relevant animal model that enables drug testing in 96-well plates while maintaining the complexity of a whole organism with physiological functions [105,113,115,139,140,141,142]. Adult and larval zPDX have the potential to capture intra-tumoral heterogeneity and to provide valuable information on the genetic, molecular and behavioral profiles of the sub-clones driving treatment resistance in patients (Figure 3 and Figure 4).

Nonetheless, each cancer avatar model is associated with drawbacks, and no one model can completely re-create human disease. All types of PDXs, including zPDX, should be carefully evaluated for their fitness to answer specific questions relevant to the patient’s tumor, including heterogeneity and sub-clone response to therapy administration. Ultimately, different models will play their own important roles across the precision medicine pipeline. For instance, larval zPDX is time-efficient in terms of guiding first-line treatment decisions, while adult zPDX, mouse PDX and organoid models could be reserved to investigate the post-treatment changes in tumor behavior and residual disease markers. Looking forward, some work is required to move zebrafish avatars closer to the clinical setting. Larval and adult zPDX methods should become standardized across laboratories so that results from avatar models are robust and reproducible. More comparisons between zPDX and mPDX models will show how these models both overlap and complement each other. Finally, more extensive co-clinical studies are needed that encompass different cancer types to compare zPDX avatar data to patient treatment responses. zPDX and matched clinical data will be essential in determining the predictiveness of zebrafish avatars to patient outcomes and the ultimate suitability of this promising model to precision cancer medicine pipelines.

## 10. Methods

Search Strategy: A comprehensive literature search was conducted using various databases such as PubMed and Google Scholar. The search was limited to articles published between 2000 and 2022. The following keywords were used in the search: “zebrafish”, “patient derived xenografts”, “intra-tumor heterogeneity”, “PDX”, “cancer avatars” and “precision medicine”.

Inclusion Criteria: Articles were included in the review if they met the following criteria: (1) They were published in peer-reviewed journals; (2) they were related to the topics of tumor heterogeneity, PDX models, the use of zebrafish in cancer research and drug screening; (3) they were published in English; and (4) they were published between 2000 and 2022.

Exclusion Criteria: Articles were excluded from the review if they did not meet the inclusion criteria.

Data Analysis: The data was analyzed by reading and summarizing the main findings of each included article and grouping them into themes or categories related to the tumor heterogeneity, existing PDX models and the application of zebrafish in biomedical research and as a cancer avatar model.

## Figures and Tables

**Table 2:** Comparison between the different avatar models.

Model	Current Status	Advantages	Disadvantages
PDO	PDO are used less frequently in terms of predicting patient outcomes compared to animal models.	Enables the study of tumor microenvironment and cell–cell interaction Medium scale drug screening due to high cost of culture Engraftment at 37 °C Animals are not required	Lacks the physiology and an integrated organ system of an in vivo model Lack of a dynamic tumor microenvironment Variable success rates for establishing the PDO
Mouse PDX	Large number of models are established and well-characterized for different cancers with high predictive ability of patient response. However, applications for the first line of therapy determination are limited due to long engraftment timelines.	Mammal Tumors generated in mPDX reflected the genomic and histologic profiles of matched patients Enables a wider range of orthotropic xenografts Engraftment at 37 °C	Weeks to months for engraftment and passaging of patient samples Variable engraftment success rates Passaging can affect the clonal composition of tumors High cost
Zebrafish PDX	Larval and adult zPDX models are established for several cancers and can predict patient treatment response. Many steps of the PDX process can be automated allowing for large-scale drug screens.	Rapid Cost effective Can be used in large-scale drug screening Transparent larvae allow for tracking of tumor cells from the moment of engraftment and imaging at single cell resolution Possibility to preserve the primary patient tumor clonal make-up	Not a mammal Optimal temperature for larval zebrafish is 28.5 °C Some orthotropic xenografts are not possible

PDX: Patient derived xenografts, PDO: Patient derived organoids.

## Data Availability

Not applicable.

## Abbreviations

5-FU	Fluorouracil
ADME	Absorption, distribution, metabolism and excretion
BMP	Bone morphogenetic protein
CEA	Carcinoembryonic antigen
CHT	Caudal hematopoietic tissues
CLSM	Confocal Laser Scanning Microscope
CML	Chronic Myeloid Leukemia
CNN	Convolutional Neural Network
COPAS	Complex Object Parametric Analysis and Sorting
CR	Complete Response
CRC	Colorectal cancer
CRLM	Colorectal Liver Metastases
CROs	Contract Research Organizations
CYP	Human cytochrome P450
DFS	Disease-free survival
dpf	Days post fertilization
ECF	5-Fluorouracil + Cisplatin + Epirubicin
ER	Estrogen receptor
FGF	Fibroblast growth factor
FLOT	Fluorouracil, leucovorin, oxaliplatin and docetaxel
FOLFIRI	5-Fluorouracil + Lederfolin + Irinotecan
FOLFOX	5-Fluorouracil + Lederfolin + Oxaliplatin
FOLFOXIRI	5-Fluorouracil + Folinic acid + Oxaliplatin + Irinotecan
FUCCI4	Fluorescent, ubiquitination-based cell cycle indicator
GBM	Glioblastoma multiforme
GC	Gastric cancer
GEM	Gemcitabine
GEM/nab-P	Gemcitabine + nab-Paclitaxel
GEMOX	Gemcitabine + Oxaliplatin
GFP	Green Fluorescent Protein
Gy	Gray
jak3	Janus kinase 3
LP	Large Particle
mPDX	mouse patient-derived xenografts
MR	Minor Response
MRI	Magnetic resonance imaging
NCI MATCH	National Cancer Institute Molecular Analysis for Therapy Choice
NSCLC	Non-Small Cell Lung Cancer
PCA	Principal component analysis
PD	Progressive Disease
PDAC	Pancreatic ductal adenocarcinoma
PDO	Patient-derived organoids
PDX	Patient-derived xenografts
PFS	Progression-free survival
PR	Progesterone receptor
PR	Partial Response
R-CNN	Regions with Convolutional Neural Network
RECIST	Response Evaluation Criteria In Solid Tumors
RTA	Relative tumor area
SD	Stable Disease
SPIM	Selective Plane Illumination Microscopy
TNBC	Triple-Negative Breast Cancer
VAST	Vertebrate Automated Screening Technology
VEGF	Vascular endothelial growth factor
WHO	World Health Organization
zPDX	Zebrafish patient-derived xenografts
